# Supplementary figures and images for: Candidatus Nitrosocaldus cavascurensis, an Ammonia Oxidizing, Extremely Thermophilic Archaeon with a Highly Mobile Genome
Source: Front Microbiol. 2018 Jan 26;9:28. doi: 10.3389/fmicb.2018.00028 (PMC5797428; doi:10.3389/fmicb.2018.00028)

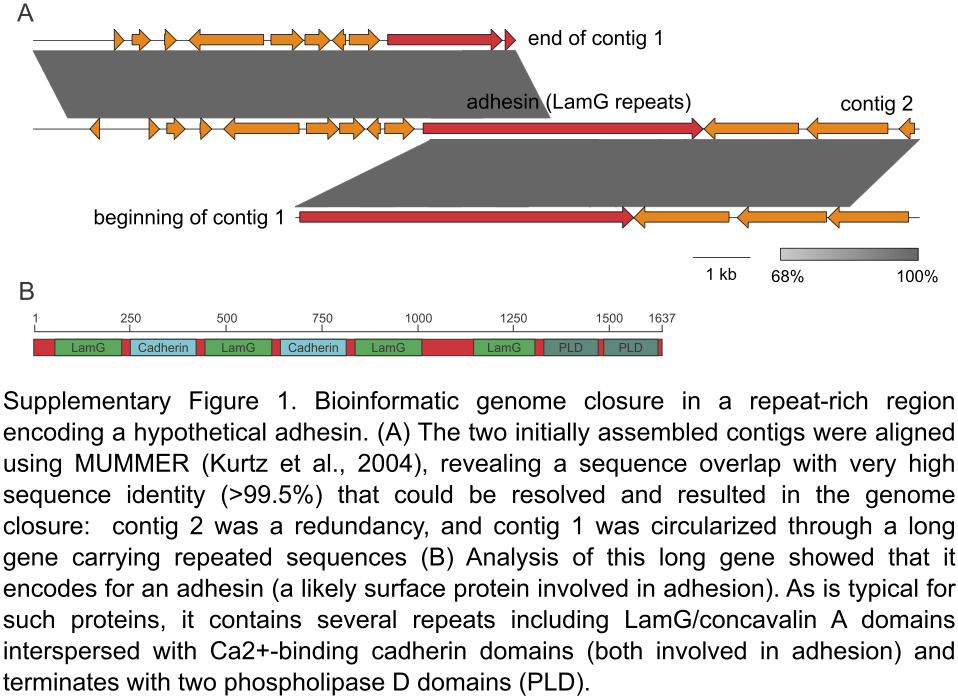

Supplement: Supplementary file 2 [file Image_1.TIFF]
