# Supplementary material for: Candidatus Nitrosocaldus cavascurensis, an Ammonia Oxidizing, Extremely Thermophilic Archaeon with a Highly Mobile Genome
Source: Front Microbiol. 2018 Jan 26;9:28. doi: 10.3389/fmicb.2018.00028 (PMC5797428; doi:10.3389/fmicb.2018.00028)
Supplement: Supplementary file 3 [file Image_2.PDF]

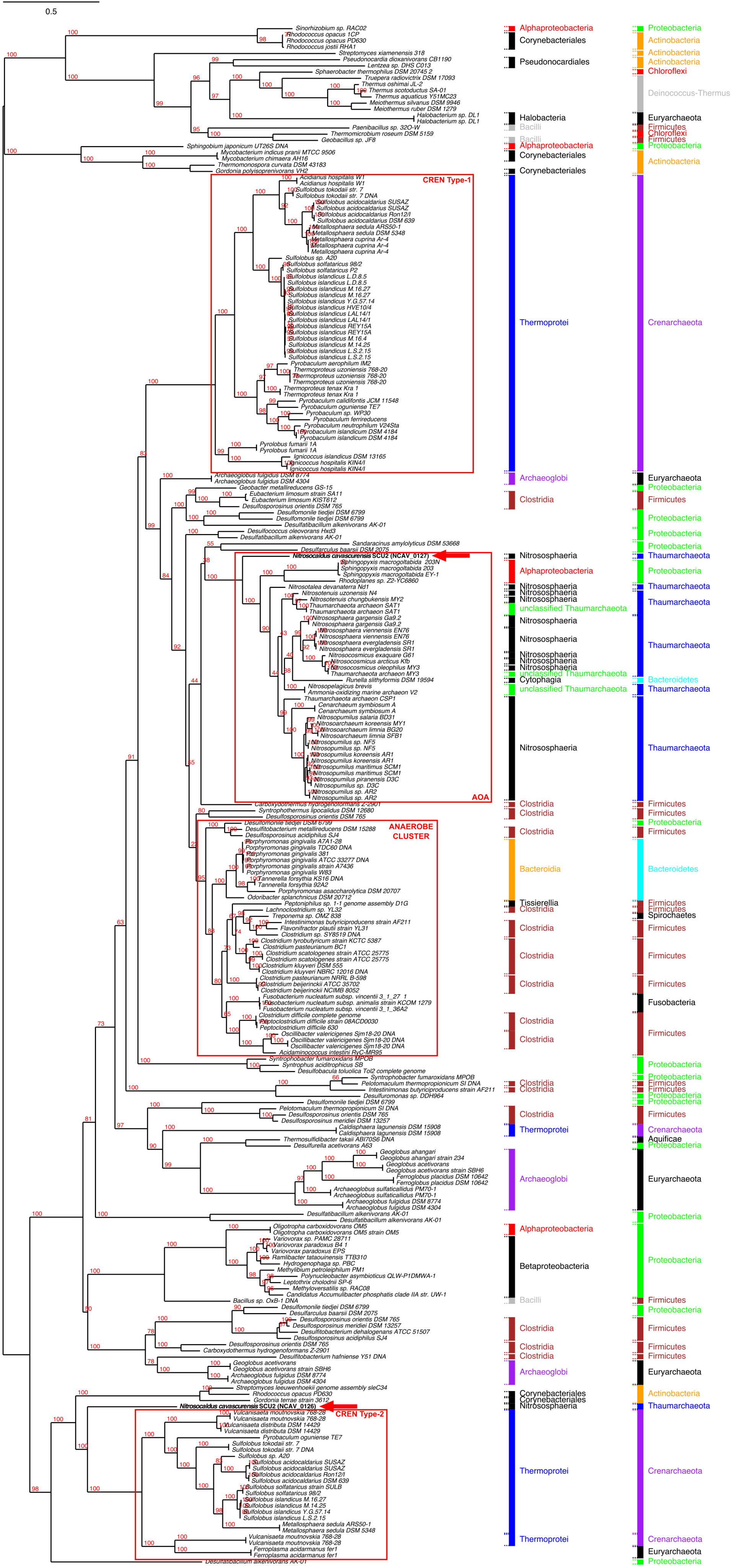

Supplementary Figure 2. Phylogenetic tree of the 4-hydoxybutyryl-CoA dehydratase protein family. A maximum likelihood phylogenetic tree was obtained with IQ-Tree v 1.5.5. The groups previously defined in (Konneke et al., 2014) "CREN type-1", "CREN type-2", "Anaerobe cluster", and "AOA" are indicated by red boxes. The organisms' class and phylum are indicated for each sequence along brackets on the right. The two homologs of this enzyme found in Ca. N. cavascurensis genome are indicated by a red arrow. This figure was generated using the Scriptree program (Chevenet et al., 2010), and the resulting SVG file modified using Inkscape.
